# Effects of Enzyme Loading and Immobilization Conditions on the Catalytic Features of Lipase From *Pseudomonas fluorescens* Immobilized on Octyl-Agarose Beads

**DOI:** 10.3389/fbioe.2020.00036

**Published:** 2020-02-28

**Authors:** Sara Arana-Peña, Nathalia S. Rios, Diego Carballares, Carmen Mendez-Sanchez, Yuliya Lokha, Luciana R. B. Gonçalves, Roberto Fernandez-Lafuente

**Affiliations:** ^1^Departamento de Biocatálisis, Instituto de Catálisis y Petroleoquímica – CSIC, Campus Universidad Autónoma de Madrid – CSIC Cantoblanco, Madrid, Spain; ^2^Departamento de Engenharia Química, Universidade Federal do Ceará, Fortaleza, Brazil

**Keywords:** lipase modulation, interfacial activation, lipase immobilization, immobilization conditions, protein intermolecular interaction

## Abstract

The lipase from *Pseudomonas fluorescens* (PFL) has been immobilized on octyl-agarose beads under 16 different conditions (varying pH, ionic strength, buffer, adding some additives) at two different loadings, 1 and 60 mg of enzyme/g of support with the objective of check if this can alter the biocatalyst features. The activity of the biocatalysts versus *p*-nitrophenyl butyrate and triacetin and their thermal stability were studied. The different immobilization conditions produced biocatalysts with very different features. Considering the extreme cases, using 1 mg/g preparations, PFL stability changed more than fourfolds, while their activities versus *p*NPB or triacetin varied a 50–60%. Curiously, PFL specific activity versus triacetin was higher using highly enzyme loaded biocatalysts than using lowly loaded biocatalysts (even by a twofold factor). Moreover, stability of the highly loaded preparations was higher than that of the lowly loaded preparations, in many instances even when using 5°C higher temperatures (e.g., immobilized in the presence of calcium, the highly loaded biocatalysts maintained after 24 h at 75°c a 85% of the initial activity, while the lowly loaded preparation maintained only 27% at 70°C). Using the highly loaded preparations, activity of the different biocatalysts versus *p*NPB varied almost 1.7-folds and versus triacetin 1.9-folds. In this instance, the changes in stability caused by the immobilization conditions were much more significant, some preparations were almost fully inactivated under conditions where the most stable one maintained more than 80% of the initial activity. Results suggested that immobilization conditions greatly affected the properties of the immobilized PFL, partially by individual molecule different conformation (observed using lowly loaded preparations) but much more relevantly using highly loaded preparations, very likely by altering some enzyme-enzyme intermolecular interactions. There is not an optimal biocatalyst considering all parameters. That way, preparation of biocatalysts using this support may be a powerful tool to tune enzyme features, if carefully controlled.

## Introduction

Lipases are among the most used enzymes at both academic and industrial level (Jaeger and Eggert, 2002; Salihu and Alam, 2015). These enzymes are reasonably stable, do not require cofactors and can catalyze a wide range of reactions (Schmid and Verger, 1998; Reetz, 2002, 2013), including promiscuous reactions (Hult and Berglund, 2007; Kapoor and Gupta, 2012), under a wide variety of reaction conditions (Brzozowski et al., 2000; Szczęsna Antczak et al., 2009; Sharma and Kanwar, 2014; Kumar et al., 2016).

Enzyme immobilization is nowadays more than a tool to solve the problem of enzyme reuse (Mateo et al., 2007; Garcia-Galan et al., 2011; Di Cosimo et al., 2013; Liese and Hilterhaus, 2013); a proper enzyme immobilization may solve many enzyme deficiencies, like enzyme stability (Iyer and Ananthanarayan, 2008; Bommarius and Paye, 2013; Stepankova et al., 2013), enzyme activity, selectivity or specificity (Rodrigues et al., 2013) inhibitions (Mateo et al., 2007) even enzyme purity may be enhanced via a well-designed immobilization protocol (Barbosa et al., 2015).

The main characteristic of lipases is that they are interfacial enzymes, that is, enzymes that are able to perform their function at the interface of drops of their natural substrates (triglycerides) (Schmid and Verger, 1998; Reis et al., 2009). This is possible by their special structure: they have one conformation where the active center is isolated from the medium by a polypeptide (lid or flat), in equilibrium with another form where this lid moves and exposes the active center to the medium (Brzozowski et al., 1991; Van Tilbeurgh et al., 1993; Verger, 1997). This form has a large and hydrophobic pocket exposed to the medium and it is the active one (Brzozowski et al., 1991; Van Tilbeurgh et al., 1993; Verger, 1997). The equilibrium between both forms may be easily modulated by the medium conditions, and in the presence of drops of a water insoluble substrate, the lipase becomes strongly adsorbed via the areas around the active center, giving a very stable form (Brzozowski et al., 1991; Verger, 1997; Carrasco-López et al., 2009). This adsorbed lipase form is in fact more stable than the enzyme in conformational equilibrium (Derewenda et al., 1994).

Due to this mechanism of action, lipases have a strong tendency to become adsorbed on any hydrophobic surface. That way they are not only adsorbed on oil drops (Beverung et al., 1999; Reis et al., 2008), but also on hydrophobic proteins (Palomo et al., 2003b; Wang et al., 2016; Zhang et al., 2017), the open form of another lipase molecule (Palomo et al., 2003a, 2004, 2005), or in any hydrophobic support (Manoel et al., 2015; Rodrigues et al., 2019). In fact, immobilization of lipases on this kind of supports is one of the most popular methods to immobilize this enzyme, as it permits the one step immobilization, purification, hyperactivation and stabilization of lipases (Bastida et al., 1998; Fernandez-Lafuente et al., 1998; Rodrigues et al., 2019). The immobilization is not a standard hydrophobic immobilization, but it is based on the interfacial activation of the lipase on the support (Manoel et al., 2015; Rodrigues et al., 2019).

The conformation of the lipase molecules is very flexible because this mobility of its structure. The features of these enzymes tend to be very sensible to changes in the reaction medium (Palomo et al., 2002). Although the immobilization using hydrophobic supports is based on weak physical interactions, it involves many groups of the enzyme, that is, the adsorption is caused by many weak enzyme-surface interactions and becomes quite strong (Rodrigues et al., 2019). The most serious problem of this immobilization protocol is the risk of enzyme release under drastic conditions or in the presence of detergent-like substrates or products (Rueda et al., 2015; Hirata et al., 2016; Rios et al., 2019c; Rodrigues et al., 2019). This may be solved using different strategies, e.g., intermolecular crosslinking (physical or chemical), use of heterofunctional supports (Rueda et al., 2015, 2016; Suescun et al., 2015; Rios et al., 2019c), trapping of the enzyme in some solid matrix (Wiemann et al., 2009; Bhattacharya et al., 2012; Nieguth et al., 2017), etc. In fact, the immobilization of lipases on hydrophobic supports via interfacial activation is used for the preparations of the most popular commercial immobilized lipase biocatalysts: Novozym 435 (Ortiz et al., 2019).

Using the lipase from *Thermomyces lanuginosus* (TLL), it was shown that depending on the pH employed in enzyme immobilization on hydrophobic resins, the enzyme regioselectivity may be altered. This permitted obtaining some preparations able to hydrolyze the position 2 of glycerides (Abreu Silveira et al., 2017) although TLL is recognized as a *sn* 1, 3 lipase (Fernandez-Lafuente, 2010). Later, using a very low enzyme load on the support (to avoid any enzyme molecule-enzyme molecule interaction) very different features of immobilized enzymes were detected (activity, specificity, stability) after immobilization on octyl-agarose under very different conditions (Lokha et al., 2020). This means that not only the structure of the lipase molecules were different under different conditions, but also that the immobilization on octyl-agarose beads was able to keep this different enzyme conformation, even though the immobilization was reversible and involved a unique mechanism and enzyme-support orientation (Rodrigues et al., 2019).

In this new study, the effect of the immobilization conditions on the final properties of the lipase from *Pseudomonas fluorescens* (PFL) has been studied. PFL is a lipase presenting a large lid and it is very used in literature (Xie, 1991; Gilbert, 1993; Rosenau and Jaeger, 2000; Rios et al., 2018; Sánchez et al., 2018) and has been used as model in different studies, like the lipase-lipase interactions that promote the formation of lipase-lipase aggregates involving two open lipase structures (Fernández-Lorente et al., 2003; Palomo et al., 2004, 2005). The comparison of the effect of the immobilization conditions on the features of the biocatalyst when using highly loaded and a lowly loaded biocatalysts is presented. Using the lowly loaded biocatalysts it may be studied how the individual immobilized lipase molecules are affected by the conditions of the immobilization. Using the highly loaded biocatalysts, it may be analyzed if the immobilization conditions may affect the enzyme-enzyme interactions (Fernandez-Lopez et al., 2017; Zaak et al., 2017b). That way, the main objective of this paper is to analyze if the changes in the immobilized enzyme properties detected using TLL can be extrapolated to other enzymes, and if enzyme-enzyme alterations can alter these effects.

Lipase immobilization on octyl-agarose beads is so fast that the enzyme molecules can pack together (Barbosa et al., 2012) and this packing may be different depending on the immobilization conditions (Fernandez-Lopez et al., 2017; Zaak et al., 2017b).

The immobilization conditions utilized in this study were selected to ensure that the enzyme was fully stable, but where some effects on lipases properties had been described in literature. That way, pH values from 5 to 9 were used. The pH during operation greatly affects the performance of this enzyme, but the immobilized enzyme is fully stable in this pH range (Rios et al., 2019b). Additives like glycerin (Lozano et al., 1994; Haque et al., 2005; Tiwari and Bhat, 2006) and increasing concentrations of NaCl (Zaak et al., 2017b), described to stabilize some lipases immobilized on hydrophobic supports were also utilized. Glycerin should favor the open form of the lipase, as it is more hydrophobic than water (that way favoring lipase immobilization (Manoel et al., 2015; Rodrigues et al., 2019) and simultaneously it should weak the enzyme-support interactions [with a negative effect on enzyme immobilization (Manoel et al., 2015; Rodrigues et al., 2019)]. The use of high ionic strength should have the contrary effect, favoring the closed form of the lipase but making the adsorption stronger. Ca^2+^ has been also used, as it can stabilize some lipases immobilized on these supports (Fernandez-Lopez et al., 2015, 2016; Arana-Peña et al., 2018; Cipolatti et al., 2018).

On the other hand, phosphate anions have been described to have a very significant destabilizing effect on lipases immobilized on these supports (Zaak et al., 2017a; Arana-Peña et al., 2018), including PFL (Rios et al., 2019c). Comparing two enzyme loads, it may be possible to determine if the enzyme-enzyme interactions can alter the effect of the immobilization conditions on the biocatalysts performance.

As hydrophobic support, octyl-agarose beads have been selected, this support has been used to immobilize PFL in many instances (Sztajer et al., 1991; Bastida et al., 1998; Fernandez-Lorente et al., 2003; Rios et al., 2019a) and agarose beads may have some applied interest (Zucca et al., 2016). PFL immobilized on this support become more stable than the enzyme covalently immobilized via multipoint attachment (Dos Santos et al., 2015).

## Materials and Methods

### Materials

*p*-Nitrophenyl butyrate (*p*NPB), triacetin and lipase from *P. fluorescens* (PFL–0.132 mg of protein/mg of powder, determined by Bradford method (1976), were purchased from Sigma-Aldrich (Spain). *R*- and *S*-methyl mandelate were from Alfa Aesar (Fisher scientific Spain). CL-4B octyl-Sepharose beads were bought from GE Healthcare (Spain). All other reagents and solvents were of analytical grade.

### Methods

All experiments were performed in triplicates and data are supplied as mean values and standard deviation.

#### Immobilization of PFL on Octyl-Agarose Beads

10 g of octyl-agarose beads was added into 100 mL of PFL solutions at the different immobilization conditions. The enzymatic solutions were prepared using 16 different immobilization buffers that differ in pH (5.0, 7.0, and 9.0) and in the presence of additives (NaCl, glycerol, CaCl_2_ or phosphate anions). The pH was adjusted after the addition of PFL into the buffer solutions. The immobilization was monitored by measuring the enzyme activity in supernatants and suspensions. The biocatalysts were produced offering 1 mg/g or 60 mg of enzyme/g of support. After immobilization of PFL, the suspension was washed thoroughly with distilled water, with 5 mM Tris at pH 7.0 and stored at 6–8°C.

#### Enzyme Activity Using Different Substrates

##### pNPB hydrolysis

The enzyme activity was determined by measuring the release of *p*-nitrophenol caused by the hydrolysis of *p*NPB. A 50 mM *p*NPB solution in acetonitrile was prepared as substrate. Therefore, 50 μL of substrate were added into 2.5 mL of 25 mM sodium phosphate buffer at pH 7.0 and 25°C. The reaction was initialized by adding 50–100 μL of enzyme (free or immobilized enzyme) and quantified at 348 nm (ε under these conditions is 5150 M^–1^ cm^–1^) (Lombardo and Guy, 1981) during 90 s, under magnetic agitation. One enzyme activity unit (U) was defined as 1 μmol hydrolyzed substrate per minute.

##### Triacetin hydrolysis

The reaction was performed using 50 mM of triacetin in 50 mM sodium phosphate buffer at pH 7.0. At this pH, the produced 1,2 diacetin suffers acyl migration and a mixture with 1,3 diacetin is obtained (Hernandez et al., 2011). 0.05–0.1 g of wet immobilized enzyme was added to the substrate solution at 25°C, under stirring. The activity of the immobilized enzyme was determined by the production of 1,2 diacetin and 1,3 diacetin, at conversion degrees between 15 and 22%. This conversion was measured using a HPLC (Kromasil C18 column of 15 cm × 0.46 cm). A solution of 15% acetonitrile-85% Milli-Q water was used as mobile phase with a flow rate of 1 mL/min. The compounds were determined with a UV detector at 230 nm and the retention times were about 4 min for both reaction products and 18 min for substrate.

##### R- or S-methyl mandelate hydrolysis

The reaction was performed using 50 mM of *R*- or *S*- methyl mandelate in 50 mM of sodium phosphate buffer at pH 7.0. A mass of 0.05–0.1 g of biocatalyst was added to the substrate solution at 25°C, under continuous stirring. The product was determined in an HPLC using a Kromasil C18 column (15 cm × 0.46 cm) with UV/VIS detector at 230 nm. A solution of 35% acetonitrile/65% Milli-Q water with 10 mM of ammonium acetate at pH 2.8 was used as mobile phase with a flow rate of 1 mL/min. Retention times for methyl mandelate and mandelic acid were 4.2 and 2.4 min, respectively.

#### Thermal Inactivations

The immobilized enzyme preparations (0.2–0.25 g) were incubated in 4–5 mL of 50 mM of Tris buffer at pH 7.0 at 70°C (lowly loaded biocatalysts) or 75°C (highly loaded biocatalysts). Periodically, samples were withdrawn and their activities were measured using *p*NPB as substrate. Residual activities were calculated as a percentage of the initial activity and half-lives were determined directly using the inactivation curves (when the activity of the biocatalyst was 50% of the initial one).

## Results and Discussion

### Preparations and Characterization of Lowly Loaded PFL Biocatalysts

#### Immobilization of PFL on Octyl-Agarose Beads Under Different Conditions

**FIGURE 1:**
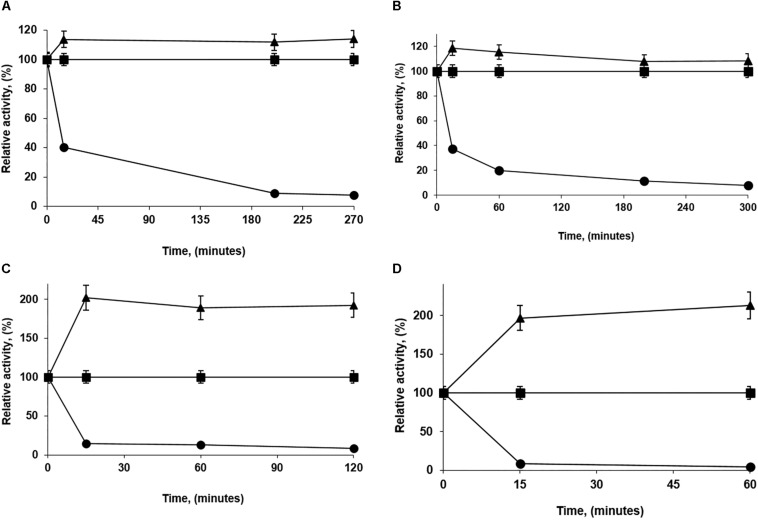
Immobilization courses of low loaded PFL preparations (1 mg/g). Immobilization conditions: **(A)** 5 mM of sodium acetate buffer with 30% glycerol at pH 5.0; **(B)** 5 mM of Tris buffer with 30% glycerol at pH 7.0; **(C)** 250 mM of sodium phosphate buffer at pH 7.0 and **(D)** 5 mM of sodium bicarbonate buffer with 250 mM NaCl at pH 9.0. Other specifications are described in section “Methods.” Solid squares: reference; solid triangles: suspension and solid circles: supernatant.

*Pseudomonas fluorescens* was immobilized under different conditions using 1 mg of enzyme/g of octyl-agarose beads. First, it was checked that the PFL activity remained unaltered after 10 h of incubation under those conditions (not shown results). Figure 1 shows some of the most extreme cases.

In all cases, the immobilization yields were fairly similar (more than 90%) and the immobilization had some positive effects on enzyme activity, although the intensity of these effects changed with the immobilization conditions. This ranged from 115 to 200% (Figure 1). Immobilization was more rapid in absence of glycerol; this reagent should slow down the immobilization but at the same time increases the percentage of individual and open forms of lipases.

#### Activity of the Different PFL Biocatalysts Versus Different Substrates

The biocatalysts were washed with 5 mM Tris at pH 7.0 and stored at 6–8°C for 1 week, to check if the changes in the features of the enzyme were permanent or can be reversed just by incubating under the same conditions for some time. Then, the activities were determined using *p*NPB, methyl mandelate and triacetin. The activity versus methyl mandelate, both *S* and *R* isomers, was too low to have a precise determination with all the biocatalysts, but the activities with the other two substrates may be found in Table 1.

**TABLE 1:** Activities versus different substrates and half-lives of different PFL biocatalysts prepared under different immobilization conditions.

**Immobilization conditions**	**Activity versus *p*NPB (U/g)**	**Activity versus triacetin (U/g)**	**Half-life (min)**
5 mM sodium acetate buffer, pH 5.0	13.25 ± 0.73	2.98 ± 0.14	90 ± 10.8
5 mM sodium acetate buffer, 100 mM NaCl, pH 5.0	13.42 ± 0.79	3.20 ± 0.17	120 ± 16.8
5 mM sodium acetate buffer, 250 mM NaCl, pH 5.0	13.47 ± 0.81	3.20 ± 0.19	140 ± 18.2
5 mM sodium acetate buffer, 30% glycerol, pH 5.0	13.83 ± 0.65	3.46 ± 0.17	110 ± 16.5
5 mM Tris buffer, pH 7.0	10.90 ± 0.62	2.68 ± 0.14	270 ± 37.8
5 mM Tris buffer, 100 mM NaCl, pH 7.0	11.67 ± 0.66	3.10 ± 0.18	180 ± 23.4
5 mM Tris buffer, 250 mM NaCl, pH 7.0	13.63 ± 0.78	3.30 ± 0.19	175 ± 22.8
5 mM Tris buffer, 30% glycerol, pH 7.0	12.79 ± 0.70	3.27 ± 0.18	240 ± 28.8
5 mM Tris buffer, 10 mM CaCl2, pH 7.0	13.86 ± 0.85	4.11 ± 0.23	280 ± 35.2
5 mM sodium phosphate buffer, pH 7.0	13.27 ± 0.77	3.42 ± 0.20	100 ± 13.6
100 mM sodium phosphate buffer, pH 7.0	13.17 ± 0.65	3.48 ± 0.18	220 ± 27.5
250 mM sodium phosphate buffer, pH 7.0	14.20 ± 0.85	3.94 ± 0.19	410 ± 60.5
5 mM sodium bicarbonate buffer, pH 9.0	13.97 ± 0.75	3.27 ± 0.16	140 ± 18.2
5 mM sodium bicarbonate buffer, 100 mM NaCl, pH 9.0	13.39 ± 0.79	3.10 ± 0.17	165 ± 22.4
5 mM sodium bicarbonate buffer, 250 mM NaCl, pH 9.0	16.29 ± 0.82	3.52 ± 0.18	155 ± 20.5
5 mM sodium bicarbonate buffer, 30% glycerol, pH 9.0	13.14 ± 0.72	2.58 ± 0.13	160 ± 21.8

*The experiments were performed using biocatalysts having 1 mg of enzyme/g of support. The activities versus *p*NPB were measured as described in section “Materials and methods” at pH 7.0 and 25°C. The inactivations were performed at 70°C using 50 mM of Tris buffer at pH 7.0. Other specifications are described in section “Methods.”*

First, the results obtained using *p*NPB will be presented. The immobilization at pH 7 (5 mM Tris) gave biocatalysts with lower activities than those immobilized at pH 5 or 9, although differences were not very large (around 20%). Increasing concentration of NaCl had a mixed effect on the immobilization. On one hand, it makes the interactions between the enzyme and the support stronger. On the other hand, the lipase molecule tends to be closed, and the only form that can be immobilized on the support is the open form (Manoel et al., 2015; Rodrigues et al., 2019). Moreover, this may alter the enzyme structure, making the exposition of internal hydrophobic groups to the medium more difficult. The increase of the NaCl concentration in the immobilization media performed at pH 5 had a very small effect on the final biocatalysts activity, while at pH 7 a progressive increase on enzyme activity was found (reaching a 20%) and at pH 9, the most active sample among the biocatalysts prepared in this paper was obtained when using 250 mM NaCl (increasing the activity of the biocatalyst immobilized at pH 9 in absence of NaCl by a 15%). The addition of 30% glycerol during the immobilization had the opposite effect on the immobilization on the support compared to the NaCl, favoring the open form while decreasing the strength of the lipase adsorption (Manoel et al., 2015; Rodrigues et al., 2019), and it can also have some effects on the enzyme structure (Lozano et al., 1994; Haque et al., 2005; Tiwari and Bhat, 2006). This additive slightly increased enzyme activity versus *p*NPB when immobilized at pH 5 (5%) and 7 (17%), while the effect was slightly negative when the enzyme was immobilized at pH 9 (6%). The addition of CaCl_2_ during the immobilization at pH 7 slightly increased the enzyme activity (near 25%), while the increase in the sodium phosphate concentration (an anion that is negative for enzyme stability) in the immobilization process at pH 7 increased the biocatalyst activity by almost 30%. That way, the difference between the most (the enzyme immobilized at pH 9 in the presence of 250 mM NaCl) and the least (PFL immobilized at pH 7 in Tris) active biocatalyst reached a value of 50% using *p*NPB as substrate.

Using triacetin as substrate, the activity of the biocatalyst prepared at pH 5 was 10% higher than that prepared at pH 7, while the immobilization of PFL at pH 9 gave again the most active biocatalyst (20% higher than immobilized at pH 7). The increase of the NaCl concentration had positive effects on the expressed activities at all pH values, using 250 mM NaCl at pH 7 the activity of the preparation increased by around 20%, when immobilized at pH 5 or 9 the increase in activity was very moderate (just around 5%). The effect of 30% glycerol was clearly positive when immobilizing the enzyme at pH 5 and 7, while it was negative when immobilizing the enzyme at pH 9 (decreasing the activity to less than 80%). The resulting catalyst was the least active biocatalyst versus triacetin among the biocatalysts that have been prepared in this paper. The use of increasing concentrations of sodium phosphate in immobilizations at pH 7 produced a significant increase in enzyme activity (by almost 50%), while the addition of CaCl_2_ produced the most active biocatalyst (almost 55% more active than the biocatalysts prepared without additives). That way, the maximum difference in enzyme activities versus triacetin was around 60% when the enzyme was immobilized at pH 7 in the presence of CaCl_2_ or when the enzyme was immobilized at pH 9 and in the presence of 30% glycerin. The effects of the different variables were similar using *p*NPB or triacetin, although the most active biocatalysts were different for each substrate.

#### Effect of the PFL Immobilization Conditions on the Biocatalysts Stability

Table 1 shows the half-lives of the different PFL preparations when incubated at 70°C and in Tris buffer at pH 7.0. As it was found using TLL (Lokha et al., 2020), the values were quite different depending on the immobilization conditions. Regarding the effect of the immobilization pH, the least stable biocatalyst was that prepared at pH 5, with a half-live around threefolds lower than that of the biocatalyst prepared at pH 7, while the immobilization at pH 9 gave better stability than the immobilization at pH 5. The presence of growing concentrations of NaCl during the immobilization improved the final enzyme stability at pH 5 (by a 55%), while immobilizing at pH 7 was negative (by 65%), and with almost no effect at pH 9.

**FIGURE 2:**
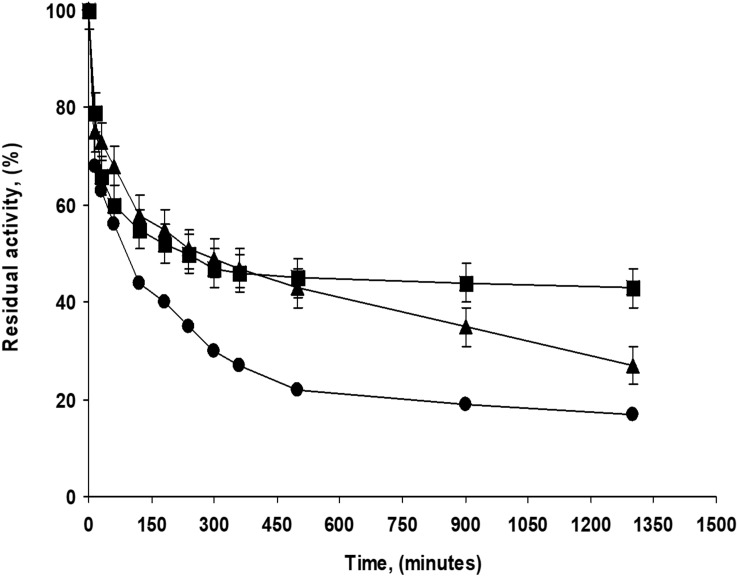
Inactivation courses of low loaded PFL preparations (1 mg/g). The biocatalysts were inactivated at 70°C, in presence of 50 mM Tris buffer, pH 7.0. Other specifications are described in section “Methods.” Solid squares: Immobilization in 5 mM buffer Tris with 30% glycerol at pH 7.0; Solid triangles: Immobilization in 5 mM buffer Tris at pH 7.0; Solid circles: Immobilization in 5 mM buffer sodium acetate at pH 5.0.

The addition of glycerin during PFL immobilization had different effects on the half-lives depending on the pH value, although the differences were negligible: at pH 5 and 9, it was slightly positive while at pH 7, the effect was slightly negative. This last biocatalyst presented a very stable fraction, the inactivation was quite multiphasic with a very stable fraction accounting for around 45% of the catalytic activity (see Figure 2). The addition of CaCl_2_ at pH 7 during immobilization did not have a significant effect on the biocatalysts half-lives. This result is surprising considering the effects using highly loaded preparations (see below). The addition of growing concentrations of sodium phosphate presented a significant and positive effect, comparing the half-live of the enzyme immobilized in 5 mM and 250 mM sodium phosphate, the difference in this parameter was more than fourfolds. That way, differences in stability ranged from a half-live of 90 min for the enzyme immobilized at pH 5 to a value of 410 min for the enzyme immobilized in the 250 mM sodium phosphate at pH 7. Figure 2 shows some inactivation courses to exemplify the differences between the biocatalysts with higher differences in their stabilities.

### Preparations and Characterization of Highly Loaded PFL Biocatalysts

#### Immobilization of PFL on Octyl-Agarose Beads

**FIGURE 3:**
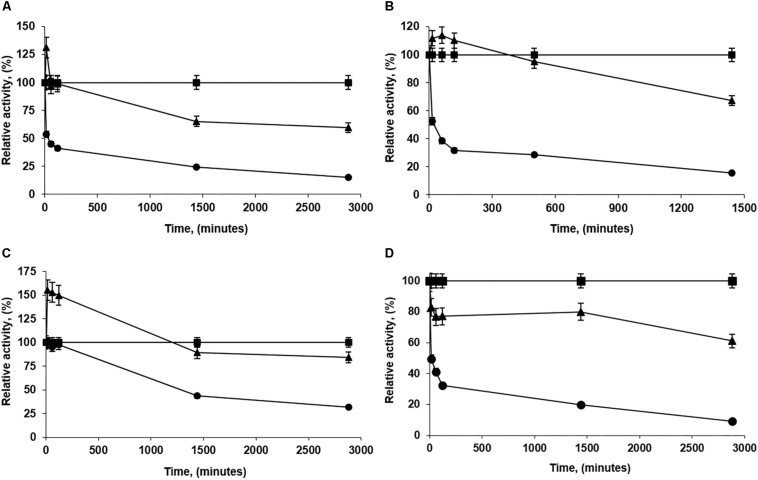
Immobilization courses of high loaded PFL preparations (60 mg/g). Immobilization conditions: **(A)** 5 mM of sodium acetate buffer at pH 5.0; **(B)** 100 mM of buffer sodium phosphate at pH 7.0; **(C)** 5 mM of Tris buffer with 30% of glycerol at pH 7.0 and **(D)** 5 mM of sodium bicarbonate buffer with 250 mM NaCl at pH 9.0. Other specifications are described in section “Methods.” Solid squares: reference; solid triangles: suspension and solid circles: supernatant.

Figure 3 shows some representative examples of the immobilization of PFL on octyl-agarose beads at a load of 60 mg/g at 16 different conditions. Table 2 shows the immobilization yield for each immobilization condition. In this instance, immobilization yield was neither identical nor almost full for all the conditions, ranging between 68 and 93%. At pH 5 and 7, the immobilization yields were fairly similar while at pH 9 the immobilization yield increased by around 8%. The increase of NaCl concentration almost did not affect the immobilization rate in all the range of studied pH values. At pH 5 and 9, the presence of glycerol slightly decreased the immobilization rate and that way also affected immobilization yields (by around 6%) while at pH 7 the immobilization rate is strongly affected, decreasing this parameter around 20% (these is the biocatalyst with the lowest enzyme loading). The presence of CaCl_2_ had no effects in the immobilization yield, while the phosphate anions decreased the immobilization yield by around 13%. This may be relevant, because the lower the immobilization rate, the lower the packing effects of the immobilization (Zaak et al., 2017b).

**TABLE 2:** Immobilization yield and final loading of the highly loaded PFL biocatalysts prepared under different conditions.

**Immobilization conditions**	**Immobilization yield (%)**	**Actual enzyme load of each biocatalysts (mg/g)**
5 mM sodium acetate buffer, pH 5.0	84.6 ± 4.2	50.8 ± 2.5
5 mM sodium acetate buffer, 100 mM NaCl, pH 5.0	79.8 ± 4.3	47.9 ± 2.6
5 mM sodium acetate buffer, 250 mM NaCl, pH 5.0	84.4 ± 4.7	50.6 ± 2.8
5 mM sodium acetate buffer, 30% glycerol, pH 5.0	79.1 ± 4.5	47.4 ± 2.7
5 mM Tris buffer, pH 7.0	86.2 ± 4.9	51.7 ± 2.9
5 mM Tris buffer, 100 mM NaCl, pH 7.0	85.1 ± 4.7	51.1 ± 2.8
5 mM Tris buffer, 250 mM NaCl, pH 7.0	84.2 ± 5.1	50.6 ± 3.0
5 mM Tris buffer, 30% glycerol, pH 7.0	68.0 ± 3.7	40.8 ± 2.2
5 mM Tris buffer, 10 mM CaCl_2_, pH 7.0	84.6 ± 4.3	50.7 ± 2.6
5 mM sodium phosphate buffer, pH 7.0	80.8 ± 5.0	48.5 ± 3.0
100 mM sodium phosphate buffer, pH 7.0	84.4 ± 4.5	50.7 ± 2.7
250 mM sodium phosphate buffer, pH 7.0	75.1 ± 3.7	45.1 ± 2.2
5 mM sodium bicarbonate buffer, pH 9.0	92.6 ± 4.2	55.6 ± 2.5
5 mM sodium bicarbonate buffer, 100 mM NaCl, pH 9.0	91.9 ± 4.3	55.1 ± 2.2
5 mM sodium bicarbonate buffer, 250 mM NaCl, pH 9.0	90.74 ± 4.6	54.4 ± 2.8
5 mM sodium bicarbonate buffer, 30% glycerol, pH 9.0	88.4 ± 3.8	53.1 ± 2.5

*Experiments were performed as described in section “Methods.”*

#### Activity With Different Substrates of Different PFL Preparations Obtained by Immobilization Under Different Conditions

The activity of the 16 preparations of PFL was evaluated with *p*NPB and triacetin (Table 3), again the activity versus methyl mandelate was too low to give reliable data (results not shown).

**TABLE 3:** Activities versus different substrates and half-lives of different PFL biocatalysts prepared under different immobilization conditions.

**Immobilization conditions**	**Activity versus *p*NPB (U/g)**	**Activity versus triacetin (U/g)**	**Half-life (min)**
5 mM sodium acetate buffer, pH 5.0	82.39 ± 4.86	310.50 ± 18.63	115 ± 15
5 mM sodium acetate buffer, 100 mM NaCl, pH 5.0	82.82 ± 4.15	347.20 ± 20.15	110 ± 14
5 mM sodium acetate buffer, 250 mM NaCl, pH 5.0	74.11 ± 4.44	296.90 ± 14.55	125 ± 16
5 mM sodium acetate buffer, 30% glycerol, pH 5.0	85.04 ± 4.42	295 ± 15.23	135 ± 17
5 mM Tris buffer, pH 7.0	72.88 ± 3.86	310.80 ± 17.52	90 ± 12
5 mM Tris buffer, 100 mM NaCl, pH 7.0	57.42 ± 3.45	281.20 ± 14.61	95 ± 13
5 mM Tris buffer, 250 mM NaCl, pH 7.0	58.10 ± 3.62	338 ± 18.42	115 ± 13
5 mM Tris buffer, 30% glycerol, pH 7.0	55.15 ± 3.52	218 ± 12.10	130 ± 17
5 mM Tris buffer, 10 mM CaCl_2_, pH 7.0	48.99 ± 3.26	289.33 ± 13.69	(84 ± 3%)*
5 mM sodium phosphate buffer, pH 7.0	54.17 ± 3.58	329.80 ± 16.85	190 ± 21
100 mM sodium phosphate buffer, pH 7.0	49.88 ± 3.11	302 ± 15.6	(59 ± 3%)*
250 mM sodium phosphate buffer, pH 7.0	49.94 ± 3.19	296 ± 14.95	(57 ± 2%)*
5 mM sodium bicarbonate buffer, pH 9.0	62.05 ± 3.08	183.10 ± 9.52	155 ± 20
5 mM sodium bicarbonate buffer, 100 mM NaCl, pH 9.0	73.94 ± 4.11	200.50 ± 9.95	170 ± 23
5 mM sodium bicarbonate buffer, 250 mM NaCl, pH 9.0	61.29 ± 3.12	184.80 ± 10.81	245 ± 29.5
5 mM sodium bicarbonate buffer, 30% glycerol, pH 9.0	79.97 ± 4.68	212.90 ± 11.01	225 ± 30.8

The experiments were performed using biocatalysts loaded using 60 mg of enzyme/g of support (see Table 2). The activities versus *p*NPB were measured as described in section “Materials and methods” at pH 7.0 and 25 °C. The inactivations were performed at 75°C using 50 mM of Tris buffer at pH 7.0. Other specifications are described in section “Methods.” * Residual activities after 24 h of inactivation.

Starting with the activity versus *p*NPB (Table 3), firstly we observe a significant decrease in enzyme specific activity, very likely caused by diffusional problems, which decreased the expected mass activity of the biocatalysts (that increased by six- to seven-folds while the enzyme loading increase 40–55) (Hamilton et al., 1974; Müller and Zwing, 1982; Berendsen et al., 2006; Shen and Chen, 2007; Fernandez-Lopez et al., 2017; Zaak et al., 2017b).

At high enzyme loading, the effect of the immobilization pH in the activity versus *p*NPB did not follow the same pattern than using low enzyme loading. Now, the highest activity is obtained when immobilizing PFL at pH 5, around 10% higher than the activity of the enzyme immobilized at pH 7 and 30% higher activity than when immobilizing at pH 9 [even when at pH 9 the immobilization yield was higher (Table 2)]. That is, now at pH 9 the biocatalyst activity was lower than when immobilizing at pH 7, in opposition to the results obtained using low loading.

At low enzyme loading, activity increased when increasing the concentration of NaCl at all pH values. Now, the effect is even negative at pH 5 and 7, and it is not significant at pH 9 (although the immobilization yield was very similar in the presence or absence of NaCl). The presence of glycerin during the immobilization at pH 7 was also negative using highly loaded preparations, while using low loading this effect was positive. This effect could be partially related to the lower immobilization yield in this instance (Table 2). At pH 5 the effect is negligible, at both enzyme loadings, while at pH 9 now the effect is positive, while at low loading was negligible (Table 3).

The presence of CaCl_2_ at pH 7 during immobilization greatly decreased the activity of the biocatalyst (by one third) while at low loading the effect of this additive was positive (Tables 1, 3). The use of growing concentration of sodium phosphate also decreased enzyme activity, while at low loading, this effect was positive (Tables 1, 3). The marginal effect of these conditions on the final immobilization yield should be considered (Table 2), that way the changes are due to real changes in intrinsic enzyme features.

When using triacetin as substrate, an increase in immobilized enzyme specific activity (per mg of immobilized enzyme) may be found in almost all cases. The expected results should be a decrease in enzyme specific activity due to diffusional substrate limitations, that can be consumed more rapidly that can penetrate inside the biocatalysts particle. This will be discussed later.

The effect of the immobilization pH on enzyme activity using triacetin as substrate is very different to that found using *p*NPB. Using highly loaded preparations, the activity at pH 5 and 7 was closely similar, while when immobilizing the enzyme at pH 9, the activity was 1.7-folds lower (Table 3) (it should be remarked that at pH 9 the immobilization yield was the highest one). This is in full disagreement with the results obtained using the lowly loaded biocatalysts, where the highest activity was obtained when immobilizing the enzyme at pH 9. The increase in NaCl concentration has neither a clear tendency nor a significant effect at the different pH values, small increases or decreases could be observed. Glycerol also presented a negligible effect when immobilizing the enzyme at pH 5 while the activity decreased when the enzyme was immobilized at pH 7 (by one third) and increased the activity by a 20% when immobilizing the enzyme at pH 9. Again, the effects were in some instances opposite to those found using low loadings, and although the enzyme loads of the biocatalysts were not identical, the differences do not explain the effect on biocatalyst activity.

The addition of CaCl_2_ at pH 7 during the immobilization had a slightly negative effect on enzyme activity, while the use of progressively higher concentration of sodium phosphate had no clear effect on enzyme activity (just a very slight decrease). Again, this does not fit the results obtained using the lowly loaded preparations.

The results, comparing highly loaded and lowly loaded, suggest that enzyme-enzyme interactions may play a very important effect on the immobilized enzyme features and on how the immobilization medium affect these enzyme properties. These effects were very clear in some cases, e.g., specific activity of the enzyme immobilized at low loading at pH 5 was around 3 U/mg but at high loading was 6 U/mg. However they were not so clear in other instances, for example when immobilizing the enzyme at pH 9 both biocatalysts exhibited almost identical specific activity (3.3 U/mg for the lowly loaded, 3.2 U/mg for the highly loaded). The fact that the specific activity versus triacetin is higher in some instances using the higher loadings is quite unexpected, because some diffusional problems may be expected using larger enzyme loadings, and this should reduce enzyme activity (Hamilton et al., 1974; Müller and Zwing, 1982; Berendsen et al., 2006; Shen and Chen, 2007; Fernandez-Lopez et al., 2017; Zaak et al., 2017b). One likely explanation could be the promotion of internal pH gradients that could increase the enzyme activity even using some phosphate in the reaction (that can act not only as buffer but as proton transporter), but the activity of immobilized PFL is slightly higher at pH 7 than at more acidic pH values even using lowly loaded preparations (not shown results). That way, this internal pH gradient can be discarded as an explanation of this improved activity. Discarding this, the most likely explanation is that enzyme-enzyme interactions occur in almost all immobilization conditions that alter the enzyme conformation and that way, the final enzyme catalytic features (e.g., enzyme activity).

**SCHEME 1:**
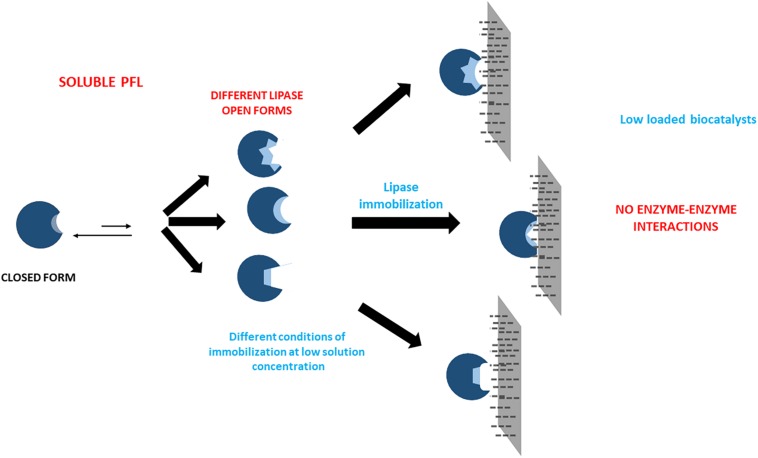
Scheme of the likely effects of immobilization conditions in the preparations of lowly loaded enzyme biocatalysts.

**SCHEME 2:**
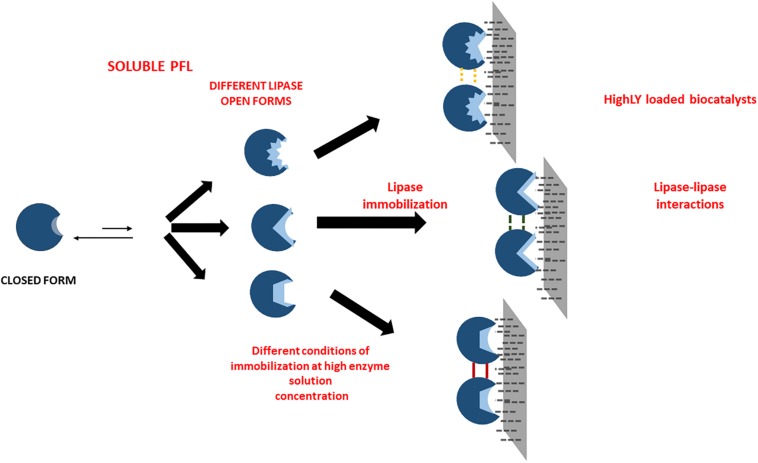
Scheme of the likely effects of immobilization conditions in the preparations of highly loaded enzyme biocatalysts.

The sometimes strong discrepancies between the effects of the immobilization conditions when using low and high loadings are another clue that suggests that protein-protein interactions may occur and greatly alter enzyme features. This is the likeliest explanation for the very significant discrepancies between both biocatalysts, and suggests the complexity of the preparation of optimal biocatalysts of PFL lipase via interfacial activation on hydrophobic support, an immobilization method apparently simple (Rodrigues et al., 2019). Using high loadings, the understanding and control of the phenomena are even more difficult than using lowly loaded biocatalysts where enzyme-enzyme interactions may be discarded. In the case of low enzyme loading, it is possible to assume that the differences of the biocatalysts prepared under different conditions are funded mainly on the fixation of different individual enzyme molecules conformations (see Scheme 1). Using high loadings, another likely explanation may join that (see Scheme 2). First, the immobilization conditions may alter the enzyme-enzyme interactions, producing other conformational changes. Moreover, the acceleration or slowdown of the immobilization process may give different distances between immobilized enzyme molecules, and this can alter the relevance of the enzyme-enzyme interactions (Zaak et al., 2017b).

#### Effect of the PFL Immobilization Conditions on the Biocatalysts Stability

Table 3 shows the half-lives of the different highly loaded biocatalyst. The inactivation experiments had to be performed at 75°C, as the enzyme stability of the highly loaded preparations was much higher than that of the lowly loaded preparations. That way, at 70°C the activity remained almost unaltered after 5 h using the highly loaded preparations. This effect of enzyme-enzyme interactions on enzyme stability had been previously described, but it is clearly visible using this enzyme (Fernandez-Lopez et al., 2017; Zaak et al., 2017b; Rios et al., 2019c).

Considering the effect of the immobilization pH, the most stable preparation was that immobilized at pH 9, and the least stable one was that immobilized at pH 7 (a 70% longer half-live when immobilized at pH 9) (Table 3). These qualitative results did not fit the values observed using low loading (Table 1) where the enzyme immobilized at pH 7 was clearly the most stable one, suggesting that enzyme-enzyme intermolecular interactions may play an important role also in enzyme stability. Increasing the concentration of NaCl at pH 5 and 7 during the enzyme immobilization, the effect on enzyme stability was not significant. However, immobilizing the enzyme at pH 9, the highest half-life was obtained when immobilizing PFL in the presence of 250 mM NaCl (by almost a 60%). The addition of glycerol during the immobilization process improved the stability at pH 7 and 9 (around 45%), and it was not significant when immobilizing the enzyme at pH 5. These results did not fit again the results when using low enzyme loading in the biocatalysts (Table 1).

The presence of CaCl_2_ during the immobilization produced a much more stable biocatalyst than the enzyme immobilized in the absence of this additive (Table 3). This occurred even though calcium has not effects when added in the inactivation solution of immobilized PFL (Rios et al., 2019c). In fact, this biocatalyst maintained over 80% of the activity after 24 h, while the biocatalysts prepared in absence of this compound presented a half-life of only 1.5 h. Using lowly loaded preparations, this positive effect was almost negligible (see Table 1). In a similar way, the presence of sodium phosphate was positive, even the use of just 5 mM permitted to improve the enzyme stability by a twofold factor compared to the enzyme immobilized in Tris buffer. Using 100 and 250 mM of this buffer during the immobilization, the biocatalyst stability became extremely high, and after 24 h it was still possible to measure almost 60% of the initial activity. Again, the positive effect of this buffer was much smaller using the lowly loaded preparation. It should be remarked that phosphate was very negative for enzyme stability when it was added in the inactivation buffer (Rios et al., 2019c). That way, predicting the effect of a variable in the final properties of the immobilized enzyme was quite difficult.

These results reinforced the strong effects of the enzyme-enzyme interactions on the features of the highly loaded biocatalysts, and the importance of the immobilization medium in tailoring these interactions.

**FIGURE 4:**
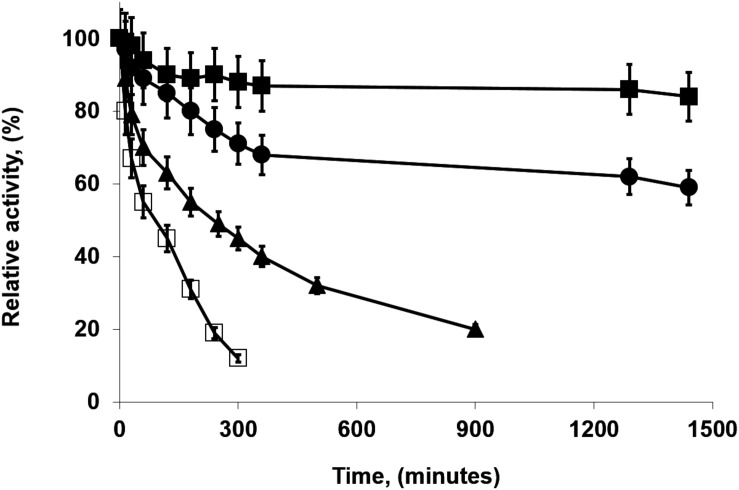
Inactivation courses of high loaded PFL preparations (60 mg/g). The biocatalysts were inactivated at 75°C, in presence of 50 mM Tris buffer, pH 7.0. Other specifications are described in section “Methods.” Solid squares: Immobilization in 5 mM Tris buffer with 10 mM CaCl_2_ at pH 7.0; Solid circles: Immobilization in 100 mM sodium phosphate buffer at pH 7.0; Solid triangles: Immobilization in 5 mM sodium bicarbonate buffer with 250 mM NaCl at pH 9.0; Empty squares: Immobilization in 5 mM Tris buffer at pH 7.0.

Figure 4 shows the inactivation at 75°C and pH 7 of some of the most and the least stable preparations. Supplementary Figure S1 shows a comparison of the inactivations of the high loaded (at 75°C) and low loaded (at 70°C) biocatalysts. The inactivations courses are almost identical when the enzyme is immobilized at pH 5 (but with 5°C of difference) while when prepared in the presence of calcium cations, the highly loaded biocatalysts was much more stable than the enzyme immobilized at low loading under similar conditions, inactivated at 5°C lower temperature.

## Conclusion

The immobilization of PFL on octyl-agarose via interfacial activation under different conditions produces biocatalysts with very different activity and stability properties. Using highly loaded preparations, the enzyme-enzyme interactions produced further changes in the enzyme features, changes that are modulated by the immobilization conditions. The modulation of the enzyme-enzyme interactions is strong enough to fully alter the effect of the immobilization conditions on enzyme properties just by altering individual enzyme molecule features. The results suggest that immobilization of lipases, at least using PFL and previously shown using TLL, via interfacial activation on hydrophobic supports must be carefully controlled, as changes in enzyme concentration, pH, additives, ionic strength, etc., may strongly affect the final immobilized enzyme performance. Once this fact is known, this PFL modulation of properties by controlling the immobilization conditions may be used as a tool to increase the library of biocatalyst, increasing that way the possibilities of finding some biocatalysts of a lipase with the desired properties.

## Data Availability Statement

All datasets generated for this study are included in the article/Supplementary Material.

## Author Contributions

SA-P, NR, DC, CM-S, and YL performed the experiments. SA-P, NR, and DC wrote the first version of the manuscript. LG and RF-L designed and supervised the experiments, and wrote the final version of the manuscript.

## Conflict of Interest

The authors declare that the research was conducted in the absence of any commercial or financial relationships that could be construed as a potential conflict of interest.

## Supplementary Material

FIGURE S1 Inactivation courses of low loaded PFL preparations (empty squares) and high loaded PFL preparations (solid squares). The low loaded PFL preparations (1 mg/g) were inactivated at 70°C, pH 7.0 and high loaded PFL preparations (60 mg/g) were inactivated at 75°C, pH 7.0. Immobilization conditions: **(A)** 5 mM of Tris HCl buffer at pH 7.0. **(B)** 100 mM of sodium phosphate buffer at pH 7.0. **(C)** 5 mM of Tris HCl buffer with 10 mM CaCl_2_ at pH 7.0. **(D)** 5 mM of sodium acetate buffer at pH 5.0.
