# Supplementary figures and images for: Effects of Enzyme Loading and Immobilization Conditions on the Catalytic Features of Lipase From Pseudomonas fluorescens Immobilized on Octyl-Agarose Beads
Source: Front Bioeng Biotechnol. 2020 Feb 28;8:36. doi: 10.3389/fbioe.2020.00036 (PMC7059646; doi:10.3389/fbioe.2020.00036)

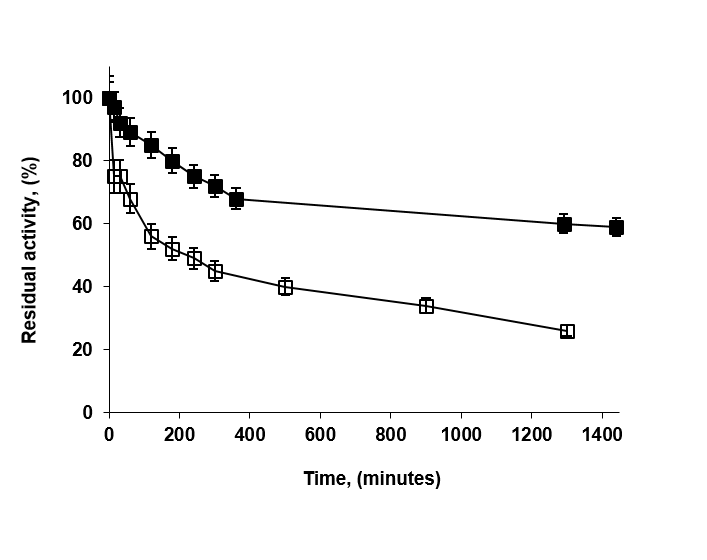

Supplement: FIGURE S1 — Inactivation courses of low loaded PFL preparations (empty squares) and high loaded PFL preparations (solid squares). The low loaded PFL preparations (1 mg/g) were inactivated at 70°C, pH 7.0 and high loaded PFL preparations (60 mg/g) were inactivated at 75°C, pH 7.0. Immobilization conditions: (A) 5 mM of Tris HCl buffer at pH 7.0. (B) 100 mM of sodium phosphate buffer at pH 7.0. (C) 5 mM of Tris HCl buffer with 10 mM CaCl2 at pH 7.0. (D) 5 mM of sodium acetate buffer at pH 5.0. [file Image_1.TIF]

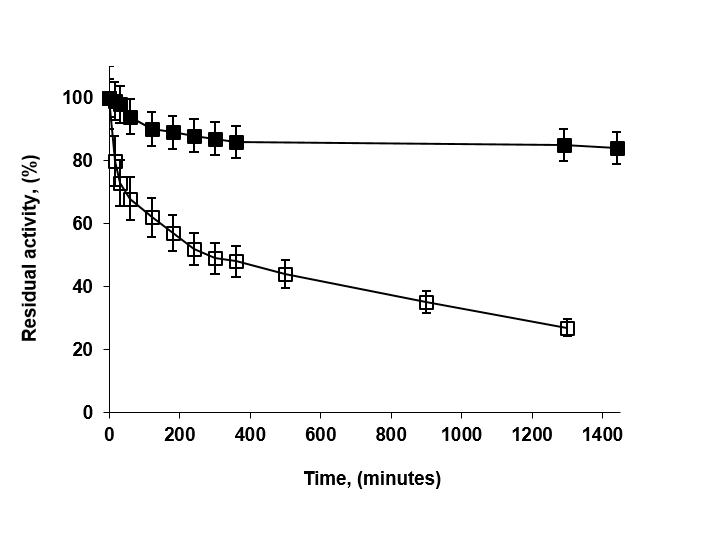

Supplement: Supplementary file 2 [file Image_2.TIF]

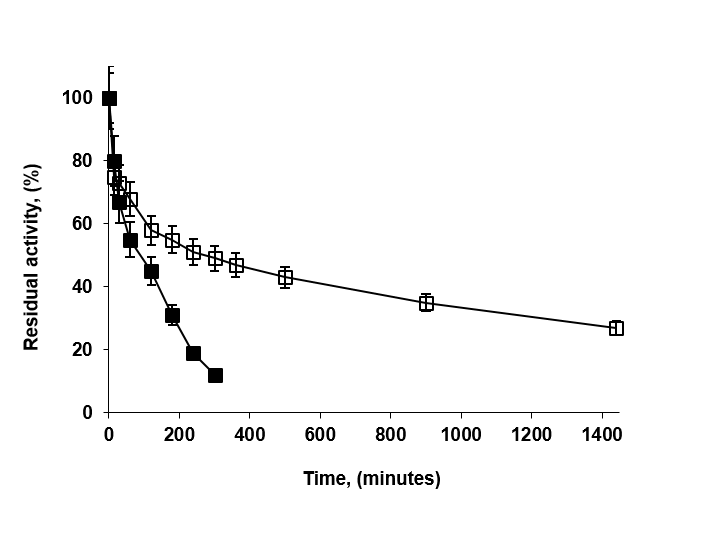

Supplement: Supplementary file 3 [file Image_3.TIF]

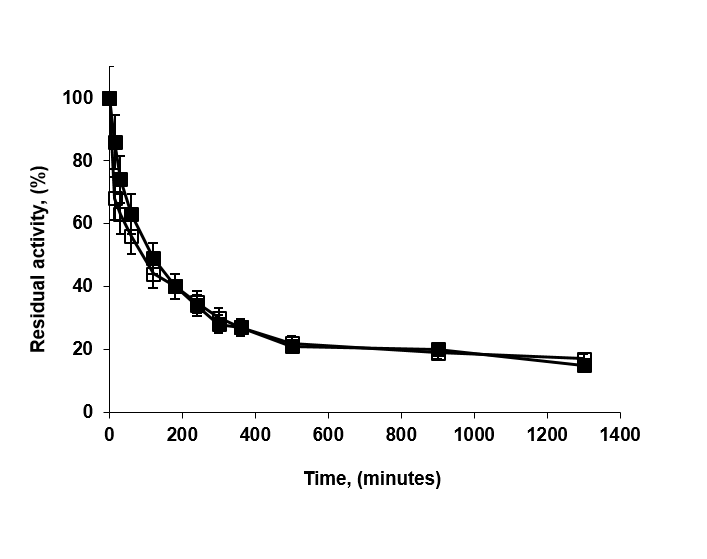

Supplement: Supplementary file 4 [file Image_4.TIF]
